# Genetic predictors of weight loss in overweight and obese subjects

**DOI:** 10.1038/s41598-019-47283-5

**Published:** 2019-07-24

**Authors:** Itziar Lamiquiz-Moneo, Rocío Mateo-Gallego, Ana M. Bea, Blanca Dehesa-García, Sofía Pérez-Calahorra, Victoria Marco-Benedí, Lucía Baila-Rueda, Martín Laclaustra, Fernando Civeira, Ana Cenarro

**Affiliations:** 1 Unidad Clínica y de Investigación en Lípidos y Arteriosclerosis, Hospital Universitario Miguel Servet, Instituto de Investigación Sanitaria Aragón (IIS Aragón), CIBERCV, Zaragoza, Spain; 2 Universidad de Zaragoza, Zaragoza, Spain

**Keywords:** Genetic markers, Obesity

## Abstract

The aim of our study was to investigate a large cohort of overweight subjects consuming a homogeneous diet to identify the genetic factors associated with weight loss that could be used as predictive markers in weight loss interventions. We retrospectively recruited subjects (N = 788) aged over 18 years with a Body Mass Index (BMI) between 25 and 40 kg/m^2^ who were treated at our lipid unit for at least one year from 2008 to 2016, and we also recruited a control group (168 patients) with normal BMIs. All participants received counselling from a nutritionist that included healthy diet and physical activity recommendations. We genotyped 25 single nucleotide variants (SNVs) in 25 genes that were previously associated with obesity and calculated genetic scores that were derived from 25 SNVs. The risk allele in *CADM2* showed a higher frequency in overweight and obese subjects than in controls (p = 0.007). The mean follow-up duration was 5.58 ± 2.68 years. Subjects with lower genetic scores showed greater weight loss during the follow-up period. The genetic score was the variable that best explained the variations in weight from the baseline. The genetic score explained 2.4% of weight change variance at one year and 1.6% of weight change variance at the end of the follow-up period after adjusting for baseline weight, sex, age and years of follow-up.

## Introduction

Obesity is one of the greatest public health problems that threatens both developed and non-developed countries. The European Health Survey indicates that in Spain, 18% of adults are obese and 37% are overweight, which is higher than the overall European obesity prevalence, which is estimated at 12%^1^. Obesity implies an excess of body fat and increases the risk of numerous comorbidities, such as cardiovascular disease (CVD), obstructive sleep apnoea, type 2 diabetes, different types of cancer and osteoarthritis, particularly in subjects with central deposition of adipose tissue^2^. It has been established that 4 million deaths worldwide can be attributed to overweight and obesity, and more than two-thirds of these deaths are due to CVD^2^. It is estimated that for each unit of increased body mass index (BMI), the risk of CVD increases by 8%^3^.

The therapeutic approach for overweight and obesity is based on lifestyle modification, prevention programmes, behavioural modification and, in extreme cases, the use of medication or bariatric surgery^4^. Life-style changes include the combination of diet modification, usually with a hypocaloric diet, and increased physical activity. However, there is a large interindividual variability in weight loss response, and not all subjects respond in the same way to the same intervention^5,6^. Although adherence is a major determinant in the response to a weight-loss intervention, a genetic component has also been recently demonstrated^7,8^. This is coherent considering that the development of obesity has a strong genetic component^9^ and that approximately 50–70% of variance in BMI is attributable to genetic differences^10^. The identification of genes that could determine the effectiveness of weight-loss strategies could lead to new approaches for the treatment and prevention of the rising pandemic of obesity.

Obesity is a heterogeneous and heritable disorder, which results from the combination among genetic susceptibility, epigenetics, metagenomics and environmental factors^11^. Monogenic syndromic forms of obesity, such as Alström syndrome, Bardet–Biedl syndrome and Cohen syndrome, have a very low frequency in the general population and are due to functional mutations in the *ALMS1*, *BBS1* or *BBS10* and *COH1* genes, respectively^12^. Monogenic or oligogenic non-syndromic forms of obesity have been described in patients with homozygous or heterozygous compound loss-of-function mutations in genes that are part of the leptin melanocortin pathway: *LEP*, *LEPR*, *POMC*, *PCSK1* and *MC4R*^13^. Reinehr *et al*. reported children who carrier *MC4R* mutations, which although they were able to lose weight with a lifestyle intervention, they had much greater difficulties in maintaining their weight loss. For instance, *MC4R* mutations led to reduced receptor function supporting the impact of these mutations on weight status^14^. However, in most cases, genetic susceptibility to obesity is due to polygenic component, which is produced by the concurrent presence of risk polymorphisms in various genes^13^. A recent genome-wide association study (GWAS) led to the discovery more than 940 independent single nucleotide variants (SNVs), which are associated with body mass index (BMI)^15^. Based on the results of all GWASs, the *FTO* gene is viewed as the main contributor to polygenic obesity in the European population^13^.

Although many studies have addressed the genetic component of obesity in the last decade, the genes associated with a differential therapeutic response to weight-loss interventions have been studied much less frequently. We aimed to explore the genetic factors that could predict weight loss by studying a large cohort of subjects with BMI >25 kg/m^2^ who were following a homogeneous dietary intervention and exercise programme and who were followed up for several years.

## Materials and Methods

### Subjects

This retrospective cohort study involved subjects attending the Lipid Unit at Hospital Universitario Miguel Servet in Zaragoza (Spain).

**Figure 1:**
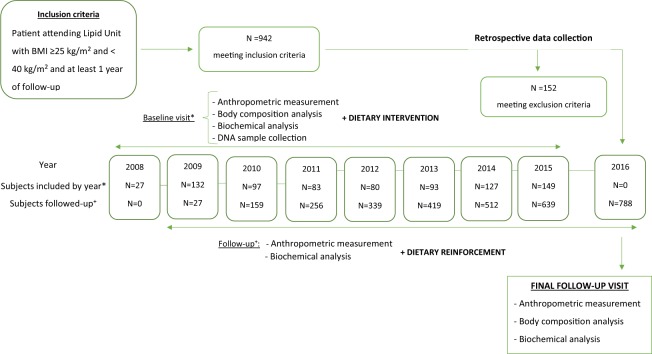
Schematic representation of the retrospective flow chart of the selection of the population for this study. BMI: Body Mass Index.

All unrelated subjects aged ≥18 years, of either sex, with a BMI between 25 and 40 kg/m^2^, who were followed-up ≥1 year and who visited the Lipid Unit at the Hospital Miguel Servet of Zaragoza from January 1^st^, 2008 to December 31^st^, 2016 were eligible for inclusion. The exclusion criteria included a personal history of malignancies, inflammatory bowel disease, bariatric surgery and taking anti-obesity drugs. Finally, 788 subjects with overweight or obesity were included (Fig. 1).

To study the SNVs associated with overweight and obesity, we randomly selected a control group of unrelated subjects with normal BMI (18.5–25 kg/m^2^) who attended the Lipid Clinic during the follow-up period. Finally, 168 control subjects were included.

All subjects signed an informed consent for a protocol that was previously approved by our local ethics committee (Comité Ético de Investigación Clínica of Aragón, Zaragoza). All the methods were performed in accordance with the relevant guidelines and regulations, and they were previously approved by our local ethics committee (Comité Ético de Investigación Clínica of Aragón, Zaragoza).

### Clinical, anthropometric, and biochemical measurements

Baseline data were collected at the first visit to the unit. Weight, height, BMI and waist circumference were measured. We also assessed body composition by bioelectrical impedance (Tanita TBF 410 GS, Omron Corporation, Tokyo, Japan), as previously described^16^. Personal and family history of CVD and other comorbidities, such as hypertension and medication use, were recorded during the clinical interview. Plasma ethylenediaminetetraacetic acid and serum samples were collected from all participants after 6 weeks without lipid-lowering drugs and after at least 10 hours of fasting. Total cholesterol and triglyceride levels were determined by standard enzymatic methods, and high-density lipoprotein cholesterol levels were measured by the oxidase method (UniCel DxC 800; Beckman Coulter Inc., Brea, California, United States), as previously described^17^. LDL cholesterol was calculated using Friedewald’s formula. Serum glucose levels were assessed by glucose-oxidase method.

A baseline dietary assessment was performed using a previously published validated food frequency questionnaire. Food and nutrient intakes were calculated as frequency x nutrient composition of particularized portion sizes, in which frequencies were divided in 9 categories (never, 1–3 times a month, 1 time a week, 2–4 times a week, 5–6 times a week, 1 time a day, 2–3 times a day, 4–6 times a day and >6 times a day) for each food item. The total energy and nutrient intakes were calculated based on previously validated Spanish food composition Tables^18^.

### Dietary intervention

All subjects received general dietary counselling based on dietary recommendations for overweight and obesity^19^ and lipid profile management according to international guidelines^20^. The dietary advice focused on increasing the intake of fruit, vegetables, whole grains, nuts, polyunsaturated fatty acids, low-fat dairy products, lean meat and fish and decreasing the consumption of saturated fatty acids, red and processed meat, soft drinks, alcohol and other sweetened products. Participants were instigated to increase their physical activity levels in consideration of their physical condition. All recommendations were provided by two expert dietitians at baseline, and they were reinforced at each follow-up visit. Each participant’s caloric prescription was represented by a deficit of 300 kcal/day, which has been calculated from energy intakes estimated. The energy intakes were estimated by multiplying the activity factor (energy expenditure for various activities established by the WHO) by the resting energy expenditure calculated with the Harris-Benedict equation.

### Follow-up

All subjects attended 3 follow-up visits during the first year and one follow-up annually thereafter. These follow-up visits included clinical, anthropometric, and lipid profile measurements and a reinforcement of the lifestyle recommendations. New diagnoses of malignancies, inflammatory bowel disease, or bariatric surgery were also exclusion criteria for the follow-up visits. Weight change was calculated using the next formula: the difference between the baseline weight and the final weight, divided by the baseline weight. Glucose change was calculated using the same formula: the difference between baseline glucose and final glucose, divided by baseline glucose. We classified the subjects into 3 categories according to weight loss: subjects who lost more than 2% of their body weight, subjects who gained more than 2% of their body weight and subjects who remained within 2% of their body weight. We chose these cut-off points to create groups with a relatively homogeneous sample size. Furthermore, we performed a subanalysis using 5% instead of 2% as a cut-off value, and this classification was used at the end of the first year and at the end of the follow-up period.

### Genetic analysis

Whole blood genomic DNA was isolated using standard methods. The SNV selection was based on previous associations with obesity in at least two independent GWASs^21–33^ and a greater than 5% frequency of the risk allele in the general population. These included the following *loci*: *BDNF*, *CADM2*, *FANCL*, *FLJ35779*, *FTO*, *GNPDA2*, *HOXC13*, *KCTD15*, *LRP1B*, *LRRN6C*, *LY86*, *MAP2K5*, *NFE2L3*, *NRXN3*, *PRKD1*, *RBJ*, *RPL27A*, *RSPO3*, *SEC*. *16B*, *SH2B1*, *TFAP2B*, *TMEM18*, *TNNI3K*, *VEGFA* and *ZNRF3-KREMEN1*. SNVs were genotyped in all subjects at the same time with TaqMan probes (Thermo Fisher) using standard methods. The *APOE* genotype was determined by DNA sequencing of exon 4, as previously described^34^. We defined the risk allele as the allele that is associated with obesity according to the GWASs that were used for SNV selection.

### Statistical analyses

Analyses were performed using SPSS version 24.0 (Chicago, Illinois, United States). The level of significance was set at *P* < 0.05. The distribution of the variables was evaluated by the Kolmogorov-Smirnov test. Quantitative variables with a normal distribution are expressed as the mean ± standard deviation and were analysed by the Student t test. Qualitative variables are expressed as percentages and were analysed by the chi-squared test. To compare the genotype and allele frequency of the genetic variants between cases and controls, we used the chi-squared test. To compare the variations in BMI throughout the follow-up period we used the paired t-test. The association of weight change with SNVs and genetic score (see below) was analysed with a linear regression model that was adjusted for baseline weight, sex, age, follow-up time, smoking status, alcohol consumption and daily consumption of vegetables, proteins, fruits, dairy products, olive oil, fish and sweets. To determine what type of model (additive, dominant or recessive) was the best, we calculated the Akaike information criterion (AIC = 2k + ln (L), where K is the number of parameters in the model and L is the likelihood) using a binary logistic regression models (yes/no) with weight loss during the follow-up as the dependent variable (Supplemental Table 1). Different regression models were used to test each inheritance. Genotypes were recorded as follows. The homozygotes for the risk allele (MM), heterozygotes (Mm), and homozygotes for the protective allele (mm) were coded to a continuous numeric variable for the genotype (as 0, 1 and 2 respectively; additive model). A dominant model was defined as contrasting genotypic groups MM + Mm vs. mm, and the recessive model was defined as contrasting genotypic groups MM vs.Mm + mm.

The baseline excess body weight, the weight that is above a normal BMI ≤25 kg/m^2^, and loss of body weight were analysed according to quartiles by the weighted genetic score (see below) using the ANOVA test.

The association of glucose change with SNVs was analysed by a linear regression model using the glucose change throughout the follow-up period as a dependent variable and adjusting the model for weight change throughout the follow-up, years of follow-up, age, sex and all SNVs. This analysis was performed after excluding subjects with type 2 diabetes.

### Genetic score

The genetic score was calculated for each subject by using the sum of the presence (for dominant or recessive model, as appropriate) of the risk genotypes in the 25 SNVs studied.

## Results

This study was conducted with a cohort that included 788 overweight patients with BMI >25 kg/m^2^ who completed at least one year of follow-up and 168 controls with BMI <25 kg/m^2^. The overweight patients were older, included a higher percentage of men and had lower HDL cholesterol levels and a higher prevalence of diabetes and hypertension than the controls. No differences in total cholesterol, triglycerides, LDL cholesterol or *APOE* genotype were found between the groups (Table 1).

**Table 1:** Baseline clinical and biochemical characteristics of subjects with BMI > 25 kg/m^2^ and subjects with BMI < 25 kg/m^2^.

		Subjects with BMI < 25 kg/m^2^ (N = 168)	Subjects with BMI > 25 kg/m^2^ (n = 788)	*p*
Age, years		48.1 ± 14.05	59.01 ± 11.64	<0.001
Men, n (%)		74 (48.7%)	470 (59.7%)	0.012
Total cholesterol, mg/dL		297 ± 47.3	294 ± 65.3	0.606
Triglycerides, mg/dL		232 ± 134	272 ± 321	0.208
LDL-cholesterol, mg/dL		205 ± 47.5	208 ± 60.4	0.228
HDL-cholesterol, mg/dL		53 ± 14.9	49.9 ± 19.6	0.008
Glucose, mg/dL		91.1 ± 25.2	98.8 ± 24.3	0.172
Diabetes, n (%)		8 (5.26%)	156 (19.8%)	<0.001
Hypertension, n (%)		24 (15.8%)	298 (37.9%)	<0.001
*APOE* genotype, n (%)	ɛ3/ɛ3	116 (70.7%)	504 (65.5%)	0.346
	ɛ3/ɛ4	34 (20.7%)	159 (20.6%)	
	ɛ3/ɛ2	9 (5.5%)	70 (9.1%)	
	ɛ2/ɛ4	1 (0.6%)	10 (1.3%)	
	ɛ4/ɛ4	4 (2.4%)	16 (2.1%)	
	ɛ2/ɛ2	0	11 (1.4%)	

Quantitative variables are expressed as the means ± standard deviations. Qualitative variables are expressed as counts (percentages). The *p* values were calculated by Student’s t test and Chi-squared test, as appropriate.

The frequency of the risk allele for obesity for each of the 25 SNVs was compared between the overweight patients and controls. The risk allele frequency of one SNV in *CADM2* was significantly higher in the overweight patients than in the controls (*p* = 0.007), and two risk alleles of SNVs in *KCTD15* and *LY86* genes were significantly lower in the overweight patients than in the controls (*p* = 0.022 and *p* = 0.047, respectively). The risk allele frequency of one SNV in the *GNPDA2* gene was significantly higher in the overweight patients than in the 1000 Genomes Project database, and the risk allele frequency of one SNV in the *RBJ* gene was significantly lower in the overweight patients than in the 1000 Genomes Project database (p = 0.014 and p < 0.001, respectively) (Supplemental Table 2). Furthermore, the frequency of the risk allele for obesity for each of the 25 SNVs was compared between the overweight and obese subjects separately and the controls. The risk allele frequency of the SNV in the *CADM2* gene was significantly higher in the obese and overweight subjects than in the controls (*p* = 0.014 and *p* = 0.001, respectively). The risk allele frequency of SNV in the *KCTD15* gene was significantly lower in overweight subjects than in controls (*p* = 0.019).

**Figure 2:**
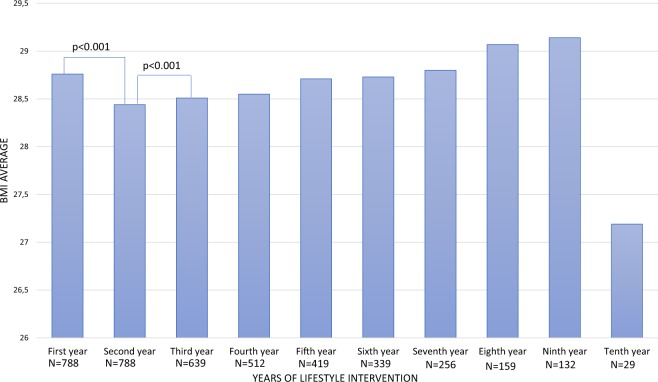
BMI variation throughout the follow-up period. BMI: Body Mass Index. The *p* value was calculated by a paired t-test.

The mean follow-up period was 5.58 ± 2.68 years (range 1.5–10 years). The dietary intervention resulted in a significant reduction of 0.68% of body weight during the follow-up (*p* = 0.003). Figure 2 indicates the body mass index values at baseline and during the follow-up in the entire population. The dietary intervention resulted in a significant reduction of 1.11% of the BMI on average during the first year of the intervention (p < 0.001). In the following years, the subjects progressively gained weight, and in the sixth year, they surpassed the baseline BMI. However, there were no significant differences in these increases in BMI, except for the interval from the second to the third year (p < 0.001) (Supplemental Table 3). Table 2 compares the baseline values and the variation in clinical and biochemical characteristics among the groups based on weight change during follow-up. Subjects who lost more than 2% of their body weight had higher baseline body weight, fat mass, abdominal visceral fat, and glucose and lower baseline cholesterol than subjects who gained more than 2% of their body weight. All subjects improved their lipid profiles during the follow-up due to lipid lowering drugs, without significant differences among the weight-change groups, except for the difference in HDL cholesterol, which increased more in subjects who lost more than 2% of their body weight. Supplemental Table 4 shows the genotype frequencies of SNVs according to the weight-change groups. Only one SNV, r10150332 in the *NRXN3* gene, showed significant differences between the groups (*p* = 0.037). Subjects who gained more than 2% weight were more frequently homozygous for the risk allele.

**Table 2:** Baseline values and variation in clinical and biochemical characteristics comparing groups based on the weight change that the subjects experienced during the follow-up.

	Subjects who lost more than 2% body weight (N = 258)	Subjects who varied by less than 2% body weight (N = 335)	Subjects who gained more than 2% body weight (N = 194)	*p*
Age, years	59.8 ± 11.9	59.4 ± 11.3	57.3 ± 11.7	0.055
Men, n (%)	145 (56.2%)	205 (61.2%)	121 (62.4%)	0.334
Baseline weight, kg	83.3 ± 13.1	79.4 ± 11.3	80.0 ± 12.8	<0.001
Baseline BMI, kg/m^2^	30.2 ± 3.37	28.4 ± 2.74	28.5 ± 2.73	<0.001
Baseline waist circumference, cm	104 ± 9.15	97.8 ± 8.75	98.7 ± 9.70	<0.001
Baseline fat mass, kg	27.3 ± 7.89	23.6 ± 6.30	24.1 ± 6.29	<0.001
Baseline muscle mass, kg	51.5 ± 10.9	50.4 ± 10.9	50.7 ± 11.5	0.736
Baseline bone mass, kg	2.73 ± 0.51	2.71 ± 0.49	2.71 ± 0.53	0.921
Baseline visceral fat level, %	11.9 ± 3.74	10.8 ± 3.52	10.8 ± 3.53	0.014
Baseline cholesterol, mg/dL	287 ± 62.1	293 ± 61.2	306 ± 74.9	0.014
Baseline triglycerides, mg/dL	281 ± 384	260 ± 271	282 ± 308	0.284
Baseline HDLc, mg/dL	49.6 ± 26.1	49.9 ± 13.7	50.1 ± 18.2	0.244
Baseline glucose, mg/dL	101 ± 27.3	96.9 ± 20.7	99.0 ± 25.7	0.007
Baseline energy consumption, Kcal/day	2299,6 ± 632	2303 ± 615	2294 ± 635	0.996
Baseline carbohydrate consumption, g/day	259 ± 91.1	256 ± 87.3	258 ± 92.1	0.983
Baseline protein consumption, g/day	94.6 ± 23.2	92.8 ± 21.8	91.8 ± 23.0	0.713
Baseline fat consumption, g/day	88.5 ± 25.5	89.6 ± 25.7	88.7 ± 26.8	0.972
Weight change after one year, %	−5.76 ± 4.06	−0.03 ± 1.14	4.60 ± 2.67	<0.001
Waist circumference change, cm	−0.88 ± 9.26	2.60 ± 6.38	5.20 ± 7.40	<0.001
Change in fat mass, %	−7.02 ± 18.1	3.65 ± 28.0	8.84 ± 20.9	<0.001
Change in muscle mass, %	0.07 ± 16.4	3.73 ± 20.3	4.52 ± 20.9	<0.001
Change in visceral fat level, %	−1.09 ± 19.1	7.32 ± 16.6	11.8 ± 17.2	<0.001
Change in cholesterol, %	−28.3 ± 19.2	−28.0 ± 22.3	−29.6 ± 21.9	0.605
Change in triglycerides, %	−13.8 ± 51.2	−13.9 ± 46.5	−5.59 ± 69.3	0.442
Change in HDLc, %	18.0 ± 132	8.99 ± 23.4	7.17 ± 27.5	0.037
Change in glucose, %	11.4 ± 77.2	8.13 ± 62.4	7.40 ± 20.4	0.241

Quantitative variables are expressed as the means ± standard deviations. The *p* values were calculated by the ANOVA test or the Kruskal-Wallis test, as appropriate.

The association between weight change and each SNV was analysed by a linear regression analysis. We categorized each SNV for recessive and dominant models for the risk allele. Five SNVs (rs7359397, rs2112347, rs29941, rs10150332, rs4929949) were significantly associated with weight change. The first one (rs7359397) was compatible with a dominant model and the rest were compatible with recessive models based on their effects. The SNVs rs2112347 and rs10150332 were significantly associated with weight change in the first year, and the other 3 SNVs were significantly associated with weight change at the end of the follow-up.

In addition, we studied the impact of all 25 SNVs included in the genetic score on weight change in the first year and during the follow-up. The percentage of weight change explained by baseline weight and the genetic score adjusted for age, gender and smoking habit was 10.1%. The genetic score explained 2.4% of weight variation, making the genetic score the variable that best explains the weight variation after baseline weight (Table 3). The percentage of weight change at the end of follow-up explained by baseline weight and genetic score adjusted for age, sex, smoking status, and follow-up duration was 8.8%. In this case, the genetic score explained 1.6% of the weight variation (Table 4). In addition, according to the U-shape relationship between time of intervention and weight loss, shown in Fig. 2, we studied the impact of the genetic score on weight loss after the first four years of the intervention. The percentage of weight change during the first four years explained by baseline weight, genetic score, and smoking status adjusted for age and sex was 12.2%. The genetic score explained 2.1% of the weight variation (Supplemental Table 5). The genetic score was significantly higher in subjects who gained more than 2% weight at the end of the follow-up than subjects who lost more than 2% weight (*p* < 0.001). To further investigate the value of the weighted genetic score, we selected subjects with changes in body weight >5%. This selection further amplified the differences in the weighted genetic score in subjects who gained over 5% of their body weight vs subjects who lost more than 5% of their body weight (scores: 18.36 vs 22.28, respectively, *p* < 0.001) (Table 5).

**Table 3:** Linear regression analysis of clinical, biochemical and genetic scores with weight change during the first year.

	β coefficient	95% CI	P	Cumulative corrected R^2^
Baseline weight	−0.001	−0.002 to −0.001	<0.001	0.077
Genetic score	0.002	0.000 to 0.004	0.026	0.101
Sex	−0.015	−0.030 to 0.000	0.048	0.101
Age	−0.023	−0.037 to 0.010	0.713	0.101
Smoking status	0.027	−0.025 to 0.040	0.675	0.101

**Table 4:** Linear regression analysis of clinical, biochemical and genetic score with weight change during the follow-up period.

	β coefficient	95% CI	P	Cumulative corrected R^2^
Baseline weight	−0.002	−0.003 to −0.001	<0.001	0.061
Genetic score	−0.004	−0.007 to −0.001	0.016	0.077
Age	0.000	−0.001 to 0.001	0.801	0.088
Sex	−0.013	−0.037 to 0.012	0.295	0.088
Years of follow-up	−0.001	−0.011 to 0.008	0.822	0.088
Smoking status	0.008	−0.004 to 0.021	0.188	0.088

95% CI, 95% confidence interval.

**Table 5:** Genetic scores of the groups based on the weight change that the subjects experienced during the follow-up.

	Subjects who lost more than 5% body weight (N = 141)	Subjects who varied by less than 5% body weight (N = 528)	Subjects who gained more than 5% body weight (N = 148)	p^1^	p^2^
Genetic score	18.36 ± 2.68	19.41 ± 3.18	22.28 ± 3.04	<0.001	<0.001
	Subjects who lost more than 2% body weight (N = 258)	Subjects who varied by less than 2% body weight (N = 335)	Subjects who gained more than 2% body weight (N = 194)	p^1^	p^2^
Genetic score	18.95 ± 2.77	19.31 ± 3.21	20.81 ± 3.56	<0.001	<0.001

Quantitative variables are expressed as the means ± standard deviations. p^1^ value calculated with the ANOVA test. *p*^2^ value calculated with Student’s t-test comparing the extreme groups.

To analyse the change in excess weight body independently of SNVs, we studied the body weight loss according to genetic score quartiles. There was no significant difference between the baseline excess body weight based on the genetic score quartile (p = 0.262). However, there were significant differences in the loss of excess body weight, in which the subjects in the lower quartile had a significantly greater loss of excess weight (p < 0.001) (Table 6**)**.

**Table 6:** Baseline excess body weight and loss of excess body weight according to the genetic score.

	First quartile (N = 173)	Second quartile (N = 185)	Third quartile (N = 275)	Fourth quartile (N = 155)	*p*
Baseline excess body weight (kg)	8.70 ± 10.02	8.07 ± 9.12	9.34 ± 10.78	7.68 ± 9.66	0.262
Loss of excess body weight (kg)	−1.47 ± 4.06	−0.90 ± 5.73	−0.77 ± 4.35	2.15 ± 4.66	<0.001

Excess body weight indicates the weight that is above a normal BMI > 25 kg/m^2^. Quantitative variables are expressed as the mean ± standard deviation. *p* values were calculated by ANOVA.

Considering the close relationship between weight and glucose, we studied the impact of the studied SNVs on glucose changes at the end of follow-up. We calculated the glucose change associated with each SNV with univariate linear regression models adjusted for age, weight change, follow-up duration, and sex. Two SNVs, rs9491696 in a recessive model and rs7359397 in a dominant model for their risk alleles, showed a statistically significant association with glucose change (*p* = 0.042 and *p* = 0.040). Weight change during the follow-up, together with follow-up duration, sex, age and these SNPs, explained up to 2.9% of the variance in fasting glucose change. Both SNVs explained 0.8% of the variance in fasting glucose change (Supplemental Table 6).

## Discussion

Obesity is a multifactorial disorder that has a genetic component but is also influenced by many environmental factors. The first genetic studies on obesity involved monogenic and extreme obesity syndromes, and the researchers focused their attention on the dysfunction of the leptin-hypothalamus pathway^35^. More recently, GWAS have allowed for the identification of many *loci* involved in interindividual weight variation that are associated with the most common expressions of obesity^15^. These association studies have contributed to the identification of new genes that are mechanistically involved in the pathophysiology of obesity^36^. In this study, we found an association between short- and long-term weight loss and genetic predisposition in a Spanish cohort. Our findings showed that subjects with higher genetic scores calculated from selected SNVs had greater weight gain, both in the short and long term, than subjects with lower genetic scores.

Among the 25 SNVs that we analysed that were previously associated with obesity, the allele frequencies of three of them, rs13078807 in the *CADM2* gene, rs29941 in the *KCTD15* gene, and rs1294421 in the *LY86* gene, were significantly different at baseline between the overweight patients and the controls. However, only rs13078807 in the *CADM2* gene showed a significantly higher prevalence of the risk allele in the overweight patients than in the controls. *CADM2* is a gene that encodes a mediator of synaptic signalling that is enriched in the brain, and *CADM2* appears to be associated with BMI in GWASs^37,38^. A previous study showed that the risk allele variant rs13078960 is associated with increased *CADM2* expression in the human hypothalamus. Deletion of Cadm2 in obese mice reduce the adiposity tissue, fasting glucose levels and improved insulin sensitivity. Therefore, *CADM2* plays a role as a potent regulator of systemic energy homeostasis^39^.

Among the 5 common genetic variants that were significantly associated with weight change, 2 of them, rs2112347 in the *FLJ35779* gene and rs10150332 in the *NRXN3* gene, were associated with weight change in the first year. At present, there is no information on the putative mechanism associating the *FLJ35779* gene with obesity^27^. In contrast, the *NRXN3* gene encodes NRXN proteins, which are located in presynaptic space and are proposed to interact with postsynaptic neuroligins. The *NRXN3* gene has been previously associated with central nervous system disorders and obesity, so its role in obesity could be a result of alterations to the nervous system^23,40^. The SNV rs29941 in the *KCTD15* gene, rs4929949 in the *RPL27A* gene and rs7359397 in the *SH2B1* gene showed an association with weight modification during the follow-up period. The *SH2B1* gene encodes an adaptor protein which increases signalling in the Janus kinase signal transducer and activator (JAK-STAT) pathway downstream of the leptin receptor^27^. Previous studies have demonstrated that natural loss-of-function mutations in the *SH2B1* gene in humans lead to both elevated food intake and severe obesity associated with leptin resistance^27^. *KCTD15* is associated with *TFAP2B*, and both of them genes have been linked to obesity in GWASs, indicating a possible physical interaction *in vivo*. Previous studies have suggested that *KCTD15* and *TFAP2B* could play a role in the pathophysiology of obesity due to the deregulation of glucose and increased peripheral resistance to insulin^41^. *RPL27A* encodes the ribosomal protein L27A, which has been related with the obesity in humans, although the molecular pathway remains unknown^41^.

The genetic score that we calculated from the sum of the 25 SNVs that were previously associated with obesity was significantly higher in patients with greater weight gain, although the weight variation explained by the score was small (2.4%). These percentage are similar to those provided by scores developed in other studies, in which the authors indicated that all BMI-associated variants combined explained 2.7% of the variation in BMI^38^. However, our genetic score is based on 25 SNVs; in contrast, the genetic score developed by Locke *et al*. was based on 97 SNVs^38^. Nevertheless, these percentages are far from the 70% of the inter-individual differences in body weight that are attributable to genetic differences between individuals^42^. Unknown genes involved in obesity, non-genetic familial influences and/or the interactions of genetic and environmental factors are probably important factors to explain this discrepancy.

Finally, we found two SNVs, rs9491696 in the *RSPO3* gene and rs7359397 in the *SH2B1* gene, that were associated with fasting glucose changes during the follow-up period. Both SNVs explained 2% of the glucose change when assessed together with weight change during the follow-up period. The expression of *SH2B1* occurs mostly in the pancreas as well as the liver, skeletal muscle and adipose tissue. Besides, SH2B1 plays a major role in insulin signalling, as might be expected given that the JAK-STAT signalling pathway is also an intracellular signalling pathway used by the insulin receptor^27^. However, *RSPO3* has only been previously associated with BMI, and its molecular pathway remains unknown^43^.

Our study has some limitations: the dietary intervention, including general counselling, was not very intense and the follow-up period included only one visit per year. However, the aim of our study was not to achieve great reductions in body weight but to study the genetic influences in subjects with overweight or obesity who were following the same dietary recommendations. The overweight and obese participants were recruited from a lipid clinic, which could be a bias in the estimation of the epidemiological or genetic influences in our study^44^. However, lifestyle is especially important in these patients, and new studies on this topic in the general population would be necessary to extrapolate our results to a healthy population. We genotyped 25 SNVs, and there are at least 940 known SNVs that are associated with BMI. However, we observe that the variance explained by our score is similar to that explained by Locke *et al*. with their score based on all 97 SNVs.

In conclusion, the genotyping of 25 SNVs that were previously associated with obesity in a grand cohort of subjects with overweight and obesity allowed for the development of a genetic score that explained 2.4% of weight change during the follow-up period. In addition, subjects with lower genetic scores showed increased weight loss during the follow-up period. However, these percentages must increase to use this genetic score as a predictive marker of weight loss. Further research is needed to fully understand the role of genetics and epigenetics in obesity, which could lead to better management and prevention of this pandemic.

## Supplementary information

Supplemental material
