# Supplementary material for: Genetic predictors of weight loss in overweight and obese subjects
Source: Sci Rep. 2019 Jul 24;9:10770. doi: 10.1038/s41598-019-47283-5 (PMC6656717; doi:10.1038/s41598-019-47283-5)
Supplement: Supplementary file 1 — Supplemental material [file 41598_2019_47283_MOESM1_ESM.docx]

**Title**

Genetic predictors of weight loss in overweight and obese subjects

**Authors**

Itziar Lamiquiz-Moneo^a^, Rocío Mateo-Gallego^a,b^, Ana M Bea^a^, Blanca Dehesa-García^a^, Sofía Pérez-Calahorra^a^, Victoria Marco-Benedí^a^, Lucía Baila-Rueda^a^, Martín Laclaustra^a^, Fernando Civeira^a,b^, Ana Cenarro^a^

**Affiliation**

^a^ Unidad Clínica y de Investigación en Lípidos y Arteriosclerosis, Hospital Universitario Miguel Servet, Instituto de Investigación Sanitaria Aragón (IIS Aragón), CIBERCV, Zaragoza, Spain

^b^ Universidad de Zaragoza, Zaragoza, Spain.

**Corresponding author**

Name: Rocío Mateo-Gallego

Address: Unidad Clínica y de Investigación en Lípidos y Arteriosclerosis, Hospital Universitario Miguel Servet, Avenida Isabel La Católica, 1-3, 50009, Zaragoza, Spain

Telephone number: (34) 976765500 (EXT 142895)

E-mail address: rmateo@unizar.es

**Supplemental table 1.** Akaike information criterion (AIC)

| SNV | Model | Ln Likelihood¹ | K^2^ | AIC^3^ |
| --- | --- | --- | --- | --- |
| rs10767664 | Additive | 1.030,811 | 5 | 1040,811 |
| rs10767664 | dummy | 1.030,798 | 6 | 1042,798 |
| rs10767664 | MM+Mm vs mm | 1030,948 | 5 | 1040,948 |
| rs10767664 | MM vs. Mm+mm | 1030,912 | 5 | 1040,912 |
| rs13078807 | Additive | 1053,46 | 5 | 1063,46 |
| rs13078807 | dummy | 1053,361 | 6 | 1065,361 |
| rs13078807 | MM+Mm vs mm | 1053,452 | 5 | 1063,452 |
| rs13078807 | MM vs. Mm+mm | 1053,398 | 5 | 1063,398 |
| rs887912 | Additive | 1042,548 | 5 | 1052,548 |
| rs887912 | dummy | 1041,47 | 6 | 1053,47 |
| rs887912 | MM+Mm vs mm | 1043,45 | 5 | 1053,45 |
| rs887912 | MM vs. Mm+mm | 1041,489 | 5 | 1051,489 |
| rs2112347 | Additive | 1057,093 | 5 | 1067,093 |
| rs2112347 | dummy | 1056,833 | 6 | 1068,833 |
| rs2112347 | MM+Mm vs mm | 1057,365 | 5 | 1067,365 |
| rs2112347 | MM vs. Mm+mm | 1056,879 | 5 | 1066,879 |
| rs1558902 | Additive | 1052,348 | 5 | 1062,348 |
| rs1558902 | dummy | 1052,32 | 6 | 1064,32 |
| rs1558902 | MM+Mm vs mm | 1052,34 | 5 | 1062,34 |
| rs1558902 | MM vs. Mm+mm | 1052,541 | 5 | 1062,541 |
| rs10938397 | Additive | 1053,8 | 5 | 1063,8 |
| rs10938397 | dummy | 1053,743 | 6 | 1065,743 |
| rs10938397 | MM+Mm vs mm | 1053,795 | 5 | 1063,795 |
| rs10938397 | MM vs. Mm+mm | 1054,152 | 5 | 1064,152 |
| rs1443512 | Additive | 1030,404 | 5 | 1040,404 |
| rs1443512 | dummy | 1030,402 | 6 | 1042,402 |
| rs1443512 | MM+Mm vs mm | 1030,42 | 5 | 1040,42 |
| rs1443512 | MM vs. Mm+mm | 1030,585 | 5 | 1040,585 |
| rs29941 | Additive | 1038,375 | 5 | 1048,375 |
| rs29941 | dummy | 1038,134 | 6 | 1050,134 |
| rs29941 | MM+Mm vs mm | 1.038 | 5 | 1048,134 |
| rs29941 | MM vs. Mm+mm | 1038,56 | 5 | 1048,56 |
| rs2890652 | Additive | 1044,191 | 5 | 1054,191 |
| rs2890652 | dummy | 1044,037 | 6 | 1056,037 |
| rs2890652 | MM+Mm vs mm | 1044,166 | 5 | 1054,166 |
| rs2890652 | MM vs. Mm+mm | 1044,1 | 5 | 1054,1 |
| rs10968576 | Additive | 978,491 | 5 | 988,491 |
| rs10968576 | dummy | 977,906 | 6 | 989,906 |
| rs10968576 | MM+Mm vs mm | 978,184 | 5 | 988,184 |
| rs10968576 | MM vs. Mm+mm | 978,61 | 5 | 988,61 |
| rs1294421 | Additive | 1018,518 | 5 | 1028,518 |
| rs1294421 | dummy | 1018,13 | 6 | 1030,13 |
| rs1294421 | MM+Mm vs mm | 1018,641 | 5 | 1028,641 |
| rs1294421 | MM vs. Mm+mm | 1018,363 | 5 | 1028,363 |
| rs2241423 | Additive | 1023,754 | 5 | 1033,754 |
| rs2241423 | dummy | 1023,751 | 6 | 1035,751 |
| rs2241423 | MM+Mm vs mm | 1026,41 | 5 | 1036,41 |
| rs2241423 | MM vs. Mm+mm | 1024,329 | 5 | 1034,329 |
| rs1055144 | Additive | 1029,846 | 5 | 1039,846 |
| rs1055144 | dummy | 1029,724 | 6 | 1041,724 |
| rs1055144 | MM+Mm vs mm | 1029,799 | 5 | 1039,799 |
| rs1055144 | MM vs. Mm+mm | 1029,858 | 5 | 1039,858 |
| rs10150332 | Additive | 1050,228 | 5 | 1060,228 |
| rs10150332 | dummy | 1046,256 | 6 | 1058,256 |
| rs10150332 | MM+Mm vs mm | 1050,73 | 5 | 1060,73 |
| rs10150332 | MM vs. Mm+mm | 1046,463 | 5 | 1056,463 |
| rs11847697 | Additive | 1045,862 | 5 | 1055,862 |
| rs11847697 | dummy | 1045,255 | 6 | 1057,255 |
| rs11847697 | MM+Mm vs mm | 1045,627 | 5 | 1055,627 |
| rs11847697 | MM vs. Mm+mm | 1046,807 | 5 | 1056,807 |
| rs713586 | Additive | 1014,64 | 5 | 1024,64 |
| rs713586 | dummy | 1014,637 | 6 | 1026,637 |
| rs713586 | MM+Mm vs mm | 1014,733 | 5 | 1024,733 |
| rs713586 | MM vs. Mm+mm | 1014,802 | 5 | 1024,802 |
| rs4929949 | Additive | 1037,503 | 5 | 1047,503 |
| rs4929949 | dummy | 1033,224 | 6 | 1045,224 |
| rs4929949 | MM+Mm vs mm | 1041,754 | 5 | 1051,754 |
| rs4929949 | MM vs. Mm+mm | 1033,447 | 5 | 1043,447 |
| rs9491696 | Additive | 1053,018 | 5 | 1063,018 |
| rs9491696 | dummy | 1053,005 | 6 | 1065,005 |
| rs9491696 | MM+Mm vs mm | 1053,13 | 5 | 1063,13 |
| rs9491696 | MM vs. Mm+mm | 1053,037 | 5 | 1063,037 |
| rs543874 | Additive | 1045,419 | 5 | 1055,419 |
| rs543874 | dummy | 1045,375 | 6 | 1057,375 |
| rs543874 | MM+Mm vs mm | 1045,383 | 5 | 1055,383 |
| rs543874 | MM vs. Mm+mm | 1046,646 | 5 | 1056,646 |
| rs7359397 | Additive | 1003,176 | 5 | 1013,176 |
| rs7359397 | dummy | 1014,637 | 6 | 1026,637 |
| rs7359397 | MM+Mm vs mm | 1002,336 | 5 | 1012,336 |
| rs7359397 | MM vs. Mm+mm | 1001,41 | 5 | 1011,41 |
| rs987237 | Additive | 1039,499 | 5 | 1049,499 |
| rs987237 | dummy | 999,147 | 6 | 1011,147 |
| rs987237 | MM+Mm vs mm | 1038,581 | 5 | 1048,581 |
| rs987237 | MM vs. Mm+mm | 1042,365 | 5 | 1052,365 |
| rs2867125 | Additive | 1032,773 | 5 | 1042,773 |
| rs2867125 | dummy | 1031,466 | 6 | 1043,466 |
| rs2867125 | MM+Mm vs mm | 1032,776 | 5 | 1042,776 |
| rs2867125 | MM vs. Mm+mm | 1032,245 | 5 | 1042,245 |
| rs1514175 | Additive | 1044,611 | 5 | 1054,611 |
| rs1514175 | dummy | 1043,756 | 6 | 1055,756 |
| rs1514175 | MM+Mm vs mm | 1045,361 | 5 | 1055,361 |
| rs1514175 | MM vs. Mm+mm | 1043,761 | 5 | 1053,761 |
| rs6905288 | Additive | 1003,242 | 5 | 1013,242 |
| rs6905288 | dummy | 1002,466 | 6 | 1014,466 |
| rs6905288 | MM+Mm vs mm | 1003,074 | 5 | 1013,074 |
| rs6905288 | MM vs. Mm+mm | 1002,921 | 5 | 1012,921 |
| rs4823006 | Additive | 683,209 | 5 | 693,209 |
| rs4823006 | dummy | 682,536 | 6 | 694,536 |
| rs4823006 | MM+Mm vs mm | 682,549 | 5 | 692,549 |
| rs4823006 | MM vs. Mm+mm | 683,942 | 5 | 693,942 |

^1^Likelihood was calculated by logistic regression model including weight loss (yes/no) during the follow-up as dependent variable, adjusted the model by length of follow-up, age, sex and baseline weight.

^2^K is the number of parameters of the model and is equal to p+1, where p is the number of predictors (excluding the intercept).

^3^Akaike information criterion (AIC) = 2K + ln(L), where L is the likelihood.

MM: Homozygotes for the major allele; Mm: heterozygotes; mm: homozygotes for the minor allele; MM+Mm vs mm: dominant model; MM vs. Mm+mm: recessive model

| Gene | Variation | Risk allele | Risk allele frequency | | | | | *p^1^* | *p^2^* | *p^3^* | *p^4^* |
| --- | --- | --- | --- | --- | --- | --- | --- | --- | --- | --- | --- |
|  |  |  | BMI <25 kg/m^2^ (N=168) | 1000 Genomes project | BMI >25 kg/m^2^ (N=788) | Obese subjects (N=249) | Overweight subjects  (N=539) |  |  |  |  |
| *BDNF* | rs10767664 | A | 0.725 | 0.770 | 0.751 | 0.758 | 0.748 | 0.345 | 0.216 | 0.297 | 0.492 |
| *CADM2* | rs13078807 | G | 0.164 | 0.209 | 0.235 | 0.237 | 0.233 | 0.007 | 0.192 | 0.014 | 0.001 |
| *FANCL* | rs887912 | T | 0.301 | 0.282 | 0.300 | 0.289 | 0.305 | 0.974 | 0.418 | 0.728 | 0.905 |
| *FLJ35779* | rs2112347 | T | 0.672 | 0.636 | 0.665 | 0.685 | 0.655 | 0.807 | 0.202 | 0.696 | 0.581 |
| *FTO* | rs1558902 | A | 0.412 | 0.435 | 0.438 | 0.444 | 0.435 | 0.400 | 0.905 | 0.374 | 0.466 |
| *GNPDA2* | rs10938397 | G | 0.493 | 0.413 | 0.481 | 0.460 | 0.492 | 0.704 | 0.014 | 0.355 | 0.956 |
| *HOXC13* | rs1443512 | A | 0.208 | 0.225 | 0.202 | 0.206 | 0.201 | 0.823 | 0.235 | 0.948 | 0.794 |
| *KCTD15* | rs29941 | G | 0.755 | 0.671 | 0.688 | 0.698 | 0.685 | 0.022 | 0.449 | 0.083 | 0.019 |
| *LRP1B* | rs2890652 | C | 0.172 | 0.166 | 0.145 | 0.131 | 0.152 | 0.221 | 0.219 | 0.113 | 0.385 |
| *LRRN6C* | rs10968576 | G | 0.267 | 0.304 | 0.265 | 0.296 | 0.251 | 0.934 | 0.072 | 0.392 | 0.566 |
| *LY86* | rs1294421 | G | 0.474 | 0.389 | 0.411 | 0.415 | 0.411 | 0.047 | 0.342 | 0.105 | 0.052 |
| *MAP2K5* | rs2241423 | G | 0.778 | 0.769 | 0.750 | 0.734 | 0.759 | 0.304 | 0.350 | 0.160 | 0.482 |
| *NFE2L3* | rs1055144 | T | 0.178 | 0.208 | 0.176 | 0.185 | 0.171 | 0.933 | 0.093 | 0.796 | 0.797 |
| *NRXN3* | rs10150332 | C | 0.164 | 0.210 | 0.182 | 0.196 | 0.175 | 0.500 | 0.132 | 0.266 | 0.666 |
| *PRKD1* | rs11847697 | T | 0.065 | 0.052 | 0.054 | 0.051 | 0.056 | 0.455 | 0.664 | 0.394 | 0.542 |
| *RBJ* | rs713568 | C | 0.500 | 0.854 | 0.483 | 0.490 | 0.481 | 0.606 | <0.001 | 0.779 | 0.561 |
| *RPL27A* | rs4929949 | C | 0.484 | 0.499 | 0.503 | 0.512 | 0.498 | 0.544 | 0.882 | 0.432 | 0.655 |
| *RSPO3* | rs9491696 | G | 0.463 | 0.459 | 0.483 | 0.486 | 0.483 | 0.522 | 0.328 | 0.536 | 0.540 |
| *SEC16B* | rs543874 | G | 0.132 | 0.188 | 0.142 | 0.145 | 0.141 | 0.649 | 0.099 | 0.616 | 0.710 |
| *SH2B1* | rs7359397 | T | 0.329 | 0.330 | 0.345 | 0.352 | 0.341 | 0.590 | 0.500 | 0.509 | 0.692 |
| *TFAP2B* | rs987237 | G | 0.170 | 0.181 | 0.179 | 0.182 | 0.179 | 0.704 | 0.910 | 0.677 | 0.729 |
| *TMEM18* | rs2867125 | C | 0.832 | 0.835 | 0.816 | 0.791 | 0.827 | 0.504 | 0.292 | 0.155 | 0.834 |
| *TNNI3K* | rs1514175 | A | 0.441 | 0.431 | 0.391 | 0.409 | 0.383 | 0.092 | 0.087 | 0.336 | 0.057 |
| *VEGFA* | rs6905288 | A | 0.625 | 0.600 | 0.590 | 0.592 | 0.589 | 0.274 | 0.672 | 0.376 | 0.274 |
| *ZNRF3-KREMEN1* | rs4823006 | A | 0.468 | 0.576 | 0.515 | 0.526 | 0.508 | 0.175 | 0.081 | 0.158 | 0.252 |

**Supplemental table 2**. Risk Allele frequency of 25 SNVs analysed.

Qualitative variables were expressed as proportion. The *p* value was calculated by Chi-squared test. *p*^1^ compared the difference between patients with BMI > 25 kg/m^2^ and patients with BMI< 25 kg/m^2^. *p*^2^ compared the difference between patients with BMI > 25 kg/m^2^ and 1000 Genomes project. *p*^3^ compared the frequencies between obese and controls. *p*^4^ compare the frequencies between overweight subjects and controls. Risk allele refers to the allele associated with obesity in the GWAS. Risk allele are from forward strand.

**Supplemental Table 3.** BMI variation throughout the follow-up.

|  | Baseline BMI (N=788) | BMI in the second year (N=788) | BMI in the third year (N= 639) | BMI in the fourth year (N=512) | BMI in the fifth year (N=419) | BMI in the sixth year (N=339) | BMI in the seventh year (N=256) | BMI in the eight year (N=159) | BMI in the ninth year (N=132) | BMI in the tenth year (N=27) |
| --- | --- | --- | --- | --- | --- | --- | --- | --- | --- | --- |
| Mean ± standard deviation | 28.76 ± 3.20 | 28.44 ± 3.12 | 28.51 ± 3.14 | 28.55 ± 3.33 | 28.71 ± 3.36 | 28.73 ± 3.30 | 28.80 ± 3.32 | 29.07 ± 3.23 | 29.14 ± 3.79 | 27.19 ± 1.88 |
| % of BMI variation compared with baseline BMI | NA | -1.11%* | -0.869% | -0.730% | -0.173% | -0.104% | +0.139% | +1.07% | +1.32% | -5.46% |
| % of BMI variation compared the previous year | NA | -1.11% * | +0.246%* | +0.140% | +0.560%⁺ | +0.069% | +0.243% | +0.938% | +0.241% | -6.692% |

Quantitative variables are expressed as means ± standard deviations. The *p* value was calculated by paired t-test. NA: No applicated. BMI: Body Mass Index. *Significantly different from baseline BMI or BMI from the previous year (p<0.001).

**Supplemental table 4**. Genotype frequencies of SNV across weight-variation groups (along the follow-up).

| Variant  *Gene* |  | Risk allele | Non-risk allele | Homozygous for the non-risk allele | Heterozygous | Homozygous for the risk allele | *p* |
| --- | --- | --- | --- | --- | --- | --- | --- |
| rs10767664  *BNDF* | Lost >2% | A | T | 14 (5.7%) | 99 (40.2%) | 133 (54.1%) | 0.493 |
|  | Variation < 2% |  |  | 24 (7.4%) | 107 (33.1%) | 192 (59.4%) |  |
|  | Gained > 2% |  |  | 14 (7.4%) | 68 (36.0%) | 107 (56.6%) |  |
| rs13078807  *CADM2* | Lost >2% | G | A | 152 (59.4%) | 88 (34.4%) | 16 (6.3%) | 0.951 |
|  | Variation < 2% |  |  | 199 (60.1%) | 107 (32.3%) | 25 (7.6%) |  |
|  | Gained > 2% |  |  | 117 (60.6%) | 64 (33.2%) | 12 (6.2%) |  |
| rs887912  *FANCL* | Lost >2% | T | C | 122 (47.4%) | 113 (44.1%) | 21 (8.2%) | 0.907 |
|  | Variation < 2% |  |  | 159 (48.8%) | 136 (41.7%) | 31 (9.5%) |  |
|  | Gained > 2% |  |  | 94 (49.0%9 | 84 (43.8%) | 14 (7.3%) |  |
| rs2112347  *FLJ35779* | Lost >2% | T | G | 29 (11.3%) | 113 (44.0%) | 115 (44.7%) | 0.656 |
|  | Variation < 2% |  |  | 42 (12.7%) | 139 (41.9%) | 151 (45.5%) |  |
|  | Gained > 2% |  |  | 29 (14.9%) | 73 (37.6%) | 92 (47.4%) |  |
| rs1558902  *FTO* | Lost >2% | A | T | 88 (34.2%) | 115 (44.7%) | 54 (21.0%) | 0.972 |
|  | Variation < 2% |  |  | 111 (33.5%) | 150 (45.3%) | 70 (21.1%) |  |
|  | Gained > 2% |  |  | 61 (31.8%) | 92 (47.9%) | 39 (20.3%) |  |
| rs10938397  *GNPDA2* | Lost >2% | G | A | 67 (26.1%) | 138 (53.7%) | 52 (20.2%) | 0.610 |
|  | Variation < 2% |  |  | 86 (26.0%) | 162 (48.9%) | 83 (25.1%) |  |
|  | Gained > 2% |  |  | 55 (28.5%) | 93 (48.2%) | 45 (23.3%) |  |
| rs1443512  *HOXC13* | Lost >2% | A | C | 165 (65.7%) | 72 (28.7%) | 14 (5.6%) | 0.719 |
|  | Variation < 2% |  |  | 204 (62.6%) | 109 (33.4%) | 13 (4.0%) |  |
|  | Gained > 2% |  |  | 121 (64.0%) | 60 (31.7%) | 8 (4.2%) |  |
| rs29941  *KCTD15* | Lost >2% | G | A | 26 (10.2%) | 108 (42.5%) | 120 (47.2%) | 0.425 |
|  | Variation < 2% |  |  | 40 (12.2%) | 131 (39.8%) | 158 (48.0%) |  |
|  | Gained > 2% |  |  | 13 (6.9%) | 83 (44.1%) | 92 (48.9%) |  |
| rs2890652  *LRP1B* | Lost >2% | C | T | 182 (71.7%) | 68 (26.8%) | 4 (1.6%) | 0.830 |
|  | Variation < 2% |  |  | 246 (74.5%) | 77 (23.3%) | 7 (2.1%) |  |
|  | Gained > 2% |  |  | 137 (72.1%) | 48 (25.3%) | 5 (2.6%) |  |
| rs10968576  *LRRN6C* | Lost >2% | G | A | 114 (48.7%) | 102 (43.6%) | 18 (7.7%) | 0.174 |
|  | Variation < 2% |  |  | 169 (54.2%) | 120 (38.5%) | 23 (7.4%) |  |
|  | Gained > 2% |  |  | 110 (61.1%) | 59 (32.8%) | 11 (6.1%) |  |
| rs1294421  *LY86* | Lost >2% | G | A | 132 (52.8%) | 19 (7.6%) | 99 (39.6%) | 0.775 |
|  | Variation < 2% |  |  | 183 (57.9%) | 22 (7.0%) | 111 (35.1%) |  |
|  | Gained > 2% |  |  | 102 (53.4%) | 15 (7.9%) | 74 (38.7%) |  |
| rs2241423  *MAP2K5* | Lost >2% | G | A | 15 (6.0%) | 95 (37.7%) | 142 (56.3%) | 0.846 |
|  | Variation < 2% |  |  | 24 (7.3%) | 112 (34.1%) | 192 (58.5%) |  |
|  | Gained > 2% |  |  | 15 (8.0%) | 67 (35.8%) | 105 (56.1%) |  |
| rs1055144  *NFE2L3* | Lost >2% | T | C | 166 (66.1%) | 79 (31.5%) | 6 (2.4%) | 0.912 |
|  | Variation < 2% |  |  | 221 (68.2%) | 95 (29.3%) | 8 (2.5%) |  |
|  | Gained > 2% |  |  | 125 (66.1%) | 61 (32.3%) | 3 (1.6%) |  |
| rs10150332  *NRXN3* | Lost >2% | C | T | 165 (64.7%) | 87 (34.1%) | 3 (1.2%) | 0.037 |
|  | Variation < 2% |  |  | 230 (69.7%) | 89 (27.0%) | 11 (3.3%) |  |
|  | Gained > 2% |  |  | 126 (65.3%) | 56 (29.0%) | 11 (5.7%) |  |
| rs11847697  *PRKD1* | Lost >2% | T | C | 225 (87.9%) | 30 (11.7%) | 1 (0.4%) | 0.636 |
|  | Variation < 2% |  |  | 297 (90.5%) | 31 (9.5%) | 0 (0%) |  |
|  | Gained > 2% |  |  | 173 (90.1%) | 18 (9.4%) | 1 (0.5%) |  |
| rs713568  *RBJ* | Lost >2% | C | T | 60 (24.4%) | 132 (53.7%) | 54 (22.0%) | 0.925 |
|  | Variation < 2% |  |  | 75 (23.1%) | 182 (56.2%) | 67 (20.7%) |  |
|  | Gained > 2% |  |  | 47 (25.4%) | 103 (55.7%) | 35 (18.9%) |  |
| rs4929949  *RPL27A* | Lost >2% | C | T | 67 (26.5%) | 123 (48.6%) | 63 (24.9%) | 0.730 |
|  | Variation < 2% |  |  | 90 (27.4%) | 151 (46.0%) | 87 (26.5%) |  |
|  | Gained > 2% |  |  | 43 (22.6%) | 92 (48.4%) | 55 (28.9%) |  |
| rs9491696  *RSPO3* | Lost >2% | G | C | 64 (24.9%) | 126 (49.0%) | 67 (26.1%) | 0.673 |
|  | Variation < 2% |  |  | 91 (27.7%) | 158 (48.2%) | 79 (24.1%) |  |
|  | Gained > 2% |  |  | 57 (29.4%) | 97 (50.0%) | 40 (20.6%) |  |
| rs543874  *SEC16B* | Lost >2% | G | A | 186 (72.1%) | 67 (26.0%) | 5 (1.9%) | 0.485 |
|  | Variation < 2% |  |  | 237 (72.9%) | 85 (26.2%) | 3 (0.9%) |  |
|  | Gained > 2% |  |  | 146 (75.6%) | 42 (21.8%) | 5 (2.6%) |  |
| rs7359397  *SH2B1* | Lost >2% | T | C | 119 (48.2%) | 90 (36.4%) | 38 (15.4%) | 0.532 |
|  | Variation < 2% |  |  | 141 (44.8%) | 131 (41.6%) | 43 (13.7%) |  |
|  | Gained > 2% |  |  | 77 (41.8%) | 82 (44.6%) | 25 (13.6%) |  |
| rs987237  *TFAP2B* | Lost >2% | G | A | 164 (64.6%) | 83 (32.7%) | 7 (2.8%) | 0.803 |
|  | Variation < 2% |  |  | 225 (68.6%) | 97 (29.6%) | 6 (1.8%) |  |
|  | Gained > 2% |  |  | 124 (64.9%) | 63 (33.0%) | 4 (2.1%) |  |
| rs2867125  *TMEM18* | Lost >2% | C | T | 12 (4.7%) | 71 (28.0%) | 171 (67.3%) | 0.561 |
|  | Variation < 2% |  |  | 8 (2.5%) | 96 (29.7%) | 219 (67.8%) |  |
|  | Gained > 2% |  |  | 9 (4.7%) | 59 (30.7%) | 124 (64.6%) |  |
| rs1514175  *TNNI3K* | Lost >2% | A | G | 94 (36.7%) | 130 (50.8%) | 32 (12.5%) | 0.409 |
|  | Variation < 2% |  |  | 124 (37.9%) | 152 (46.5%) | 51 (15.6%) |  |
|  | Gained > 2% |  |  | 70 (36.6%) | 85 (44.5%) | 36 (18.8%) |  |
| rs6905288  *VEGFA* | Lost >2% | A | G | 45 (18.3%) | 120 (48.8%) | 81 (32.9%) | 0.786 |
|  | Variation < 2% |  |  | 57 (18.3%) | 138 (44.2%) | 117 (37.5%) |  |
|  | Gained > 2% |  |  | 31 (16.6%) | 87 (46.5%) | 69 (36.9%) |  |
| rs4823006  *ZNRF3-*KREMEN1 | Lost >2% | A | G | 34 (20.7%) | 90 (54.9%) | 40 (24.4%) | 0.989 |
|  | Variation < 2% |  |  | 48 (21.6%) | 119 (53.6%) | 55 (24.8%) |  |
|  | Gained > 2% |  |  | 28 (22.6%) | 68 (54.8%) | 28 (22.6%) |  |

Qualitative variables were expressed as count (percentage). The *p* value was calculated by Chi-squared test. Allele are from the forward strand.

**Supplemental table 5**. Lineal regression analysis of clinical, biochemical and SNVs with weight change during the first 4 years (N=488).

|  | β Coefficient | 95% CI | P | Corrected R^2^ |
| --- | --- | --- | --- | --- |
| Baseline weight | -0.121 | -0.176 to -0.066 | <0.001 | 0.077 |
| Smoking status | 0.998 | 0.246 to 1.756 | 0.009 | 0.101 |
| Genetic score | 0.252 | 0.058 to 0.446 | 0.011 | 0.122 |
| Age | 0.035 | -0.027 to 0.097 | 0.272 | 0.122 |
| Sex | -0.445 | -1.996 to 1.107 | 0.572 | 0.122 |

95%CI: 95% confidence interval

**Supplemental table 6**. Lineal regression analysis of clinical, biochemical and SNVs with glucose change along the follow-up.

|  | β Coefficient | 95% CI | P | Corrected R^2^ |
| --- | --- | --- | --- | --- |
| Weight change along the follow-up | 32.184 | 9.853 to 54.514 | 0.005 | 0.012 |
| Years of follow-up | 0.753 | 0.226 to 1.279 | 0.005 | 0.021 |
| rs7359397 as dominant model | 2.962 | 0.141 to 0.57821 | 0.040 | 0.026 |
| rs9491696 as recessive model | 3.234 | 0.007 to 6.461 | 0.042 | 0.029 |
| Age | -0.016 | -0.141 to 0.109 | 0.806 | 0.029 |
| Sex | -1.590 | -4.521 to 1.342 | 0.287 | 0.029 |

95%CI: 95% confidence interval
